# Amble Gait EEG Points at Complementary Cortical Networks Underlying Stereotypic Multi-Limb Co-ordination

**DOI:** 10.3389/fnhum.2021.691482

**Published:** 2021-08-03

**Authors:** Joyce B. Weersink, Natasha M. Maurits, Bauke M. de Jong

**Affiliations:** Department of Neurology, University Medical Center Groningen, University of Groningen, Groningen, Netherlands

**Keywords:** amble gait, EEG, multi-limb coordination, anti-phase, arm swing, supplementary motor area, right hemisphere, event related spectral perturbations

## Abstract

**Background:**

Walking is characterized by stable antiphase relations between upper and lower limb movements. Such bilateral rhythmic movement patterns are neuronally generated at levels of the spinal cord and brain stem, that are strongly interconnected with cortical circuitries, including the Supplementary Motor Area (SMA).

**Objective:**

To explore cerebral activity associated with multi-limb phase relations in human gait by manipulating mutual attunement of the upper and lower limb antiphase patterns.

**Methods:**

Cortical activity and gait were assessed by ambulant EEG, accelerometers and videorecordings in 35 healthy participants walking normally and 19 healthy participants walking in amble gait, where upper limbs moved in-phase with the lower limbs. Power changes across the EEG frequency spectrum were assessed by Event Related Spectral Perturbation analysis and gait analysis was performed.

**Results:**

Amble gait was associated with enhanced Event Related Desynchronization (ERD) prior to and during especially the left swing phase and reduced Event Related Synchronization (ERS) at final swing phases. ERD enhancement was most pronounced over the putative right premotor, right primary motor and right parietal cortex, indicating involvement of higher-order organization and somatosensory guidance in the production of this more complex gait pattern, with an apparent right hemisphere dominance. The diminished within-step ERD/ERS pattern in amble gait, also over the SMA, suggests that this gait pattern is more stride driven instead of step driven.

**Conclusion:**

Increased four-limb phase complexity recruits distributed networks upstream of the primary motor cortex, primarily lateralized in the right hemisphere. Similar parietal-premotor involvement has been described to compensate impaired SMA function in Parkinson’s disease bimanual antiphase movement, indicating a role as cortical support regions.

## Introduction

Walking is characterized by a stereotypic multi-limb movement pattern with stable phase relations between all four limbs (Wannier et al., 2001). This does not only hold for quadripedal gait, but also for upright human gait in which antiphase arm swing is opposite to the antiphase movements of the legs. Stability of such stereotypic movement pattern is speed-related with abrupt transitions from e.g., a stable antiphase to a stable in-phase pattern at higher speed, which is seen in transitions both from quadrupedal trot to gallop and from bimanual antiphase to in-phase movement (Kelso, 1984). One may consider that such transition at increasing speed represents the maintenence of stability by a simpler movement pattern serving energetic efficiency (Tuller and Kelso, 1989; Carson, 1995). In conditions of pathology, enhanced complexity of antiphase movements finds support by the observed difficulty of particularly making bimanual anti-phase movements that occurs e.g., in Parkinson’s disease when patients tend to revert anti- to in phase movements (Johnson et al., 1998; Almeida et al., 2002). This is also consistent with the increase of mirror movements of opposite hands as a consequence of impaired transcallosal inhibition (Welniarz et al., 2019). The regularity of these patterns and abrupt phase transitions provide arguments for the involvement of a central pattern generator (Kelso, 1984). Neuronal circuitries that may generate such rhythms have been identified at levels of spinal cord and brain stem, and are strongly embedded in wider distributed circuitries, including cerebral cortical regions. This logically serves the dynamic involvement of sensory modalities and cognition in gait control (Takakusaki, 2017).

In addition to the antiphase pattern of opposite limbs in human walking, the upper and lower limb of the same side of the body move in antiphase too. The latter resembles antiphase movement of the unilateral fore- and hindleg in the majority of quadripeds. In contrast, amble gait in e.g., camels is characterized by in-phase movementsof the two limbs on the same side, which particularly occurs in trot. These well-coordinated four-limb movement patterns serve to maintain stability and energetic efficiency during the dynamics of locomotion (Ortega et al., 2008; Umberger, 2008; Yizhar et al., 2009; Bruijn et al., 2010; Meyns et al., 2013), also in human gait (Weersink et al., 2019). In humans, amble gait would imply that for both the upper and lower limbs an antiphase movement pattern is maintained, but that the arm and leg on the same side of the body move in an in-phase mode. The comparison between opposite antiphase movements in the natural condition of normal gait and the experimantal setting of amble gait thus provides an opportunity to explore dynamic qualities of cerebral activity associated with multi-limb phase relations in human gait.

The Supplementary Motor Area (SMA) plays an important role in bimanual coordination, with a stronger contribution to antiphase than to in-phase movements of the opposite hands (Stephan et al., 1999). This is consistent with the observation that lesions of the SMA may lead to increased mirror movements (Brinkman, 1981; Potgieser et al., 2014). In addition to the contribution of the SMA, increasing complexity of bimanual co-ordination, implicated in e.g., antiphase relative to in-phase movement, appears to demand a wider distributed ciruitry, including premotor and parietal regions (Swinnen, 2002; Debaere et al., 2004; Wenderoth et al., 2004). Particularly the right dorsal premotor cortex has been demonstrated to contribute to antiphase movements of the two hands (Sadato et al., 1997; de Jong et al., 2002). While overlap of circuitries involved in antiphase movements of either hands or feet highlighted involvement of the right dorsal premotor cortex in association with a contribution of the right anterior parietal cortex, the SMA was not seen in such overlap (de Jong et al., 2002). This is consistent with the finding that no increase of SMA activation was seen in bipedal antiphase movement compared to in-phase movement of the feet. Indeed, the role of the SMA in specifically antiphase movement of the feet is not well established. This may seem at odds with the previously described role of the SMA in human gait (Della Sala et al., 2002; Weersink et al., 2019). However, it should be considered that in gait, antiphase movements are generated by proximal limb muscles while the referred experimental paradigms concerned distal limb movements. The concept that one of the SMA contributions to gait control concerns the effective recruitment of arm swing is supported by the association between reduced SMA activity and (i) the experimental condition of gait without arm swing and (ii) the circumstance of Parkinson’s disease (PD), a disease characterized by small-step walking with reduced or absent arm swing (Jenkins et al., 1992; Jahanshahi et al., 1995; Sabatini et al., 2000; Weersink et al., 2019, 2020). The fact that the instruction to start walking with enhanced arm swing results in an improvement of gait initiation in PD patients, associated with virtual normalization of SMA activity, further supports the idea that upper-limb antiphase movement intrinsically serves efficient gait control, mediated by the SMA (Weersink et al., 2020).

The above referred overlap of activations related to antiphase movement of either hands or feet, that was seen in the right premotor and anterior parietal cortex of healthy subjects, without involvement of the SMA, hints at a resemblance with the cerebral pattern demonstrated in PD patients when performing manual antiphase movements (Wu et al., 2010). While no increase of SMA activity was seen when these patients made such antiphase movements, compared to in-phase movements, activity increases did occur in parietal and premotor cortical regions, of which the right premotor and anterior parietal cortex showed stronger functional connections with the impaired SMA. These observations in healthy subjects and in PD patients underscore the presence of wider distributed cortical circuitry specifically involved in antiphase movement patterns, and suggest that distinct SMA-connected circuitry may serve functional compensation of impaired SMA function. Given such coherent function of the SMA and these interconnected regions, we considered that by manipulating the mutual attunement of the upper and lower limb antiphase patterns, e.g., by introducing the experimental condition of amble gait, a complementary circuitry related to antiphase movement might be challenged, either with or without increased activity of the SMA.

For recording cortical activity during overground walking, ambulant electroencephalography (EEG) can be employed. Analysis of event related spectral perturbations (ERSP) in the EEG enables the assessment of average dynamic changes in power across the frequency spectrum as a function of time relative to successive events of the gait cycle. Alpha (7–12 Hz), beta (12–30 Hz) and gamma (30+ Hz) oscillations appear to reflect strong movement-related modulations within the motor system (Ohara et al., 2001; Gross et al., 2005; Pollok et al., 2005; Cheyne et al., 2008; Houweling et al., 2008). In general, decreases of oscillations in alpha and beta band occur prior to and during movement (i.e., Event Related Desynchronization (ERD)) and are followed by a post-movement rebound of Event Related Synchronization (ERS) (Pfurtscheller and Lopes da Silva, 1999; Klimesch et al., 2007; Engel and Fries, 2010). In gait, a within-step pattern of activation and deactivation has been demonstrated over the sensorimotor cortex (Gwin et al., 2011; Wagner et al., 2014; Weersink et al., 2019). Particularly the cyclic pattern of hemispheric midline modulations within the low-gamma band, indicative for involvement of the SMA, has been related to the organization of active walking (Wagner et al., 2012; Seeber et al., 2014; Weersink et al., 2019).

The main aim of the present study was to gain insight in dynamical qualities of cortical circuitry implicated in the antiphase movement patterns of opposite limbs in human gait, when challenged by increasing the complexity of these stereotypic patterns. To that end, the experimental condition of amble gait was introduced, in which antiphase movements of the opposite limbs remained the same while ipsilateral movements were performed in an in-phase pattern. Cortical activity was assessed by ERSP analysis of ambulant EEG recordings while accelerometry recordings enabled identification of gait phases in the EEG and calculation of gait characteristics. In this way, we were able to address the questions whether the increased complexity of tuning the upper and lower antiphase patterns in amble gait (i) demands increased activity of specifically the SMA (ii) is associated with increased premotor and parietal activity of particularly the right hemisphere or (iii) requires conjoined increase of parietal—premotor and SMA activity. The answers to these questions were expected to provide more insight in the baseline and potentially compensatory cortical circuitries involved in multi-limb phase relations in healthy and neurologically impaired human gait.

## Materials and Methods

### Participants

35 healthy participants (17 males and 18 females, median age 67 ± 9 years) were included in this study. Their advanced age enabled future reference with patients suffering from neurodegenerative diseases such as Parkinsons disease. None of the participants suffered from neurological disorders, used medication that influenced movement or had cognitive problems (median MMSE 29 ± 1). All participants were right handed according to the Annett Handedness scale (Annett, 1970) and gave their written informed consent. The study was executed according to the Declaration of Helsinki (2013) and was approved by the medical ethical committee of the University Medical Center Groningen.

### Task and Experimental Set-Up

The experiment consisted of two sessions conducted consecutively on the same day with a small break in between. Participants were instructed to walk at their own preferred speed through a hallway of 150 m in a straight line from start to finish and back. During the baseline condition, they were asked to walk as they would do when taking a walk in the park. Nineteen participants (9 males and 10 females, 69 ± 4 years) also performed a second session that consisted of amble gait where they were instructed to walk while swinging their arms in-phase with their legs. Prior to this condition, participants practiced this condition until approval of the investigator. Afterward, video recordings were used to check whether participants did not accidently swing their arms in antiphase with their legs. The amble gait condition always followed the baseline condition to avoid that the patients would become highly aware of their arm swing with the risk of influencing natural gait. The study comprises of data collected in two different periods; the study design during the first period of data collection (*n* = 16) did not include the amble gait condition, but data were included to provide a more reliable average of the ERSP plots for future comparison purposes.

During the two sessions, ambulant monopolar EEG was recorded using a cap with 32 active Ag-AgCl electrodes (EasyCap GmbH, Herrsching, Germany) located according to the international 10–20 system. These active electrodes can considerably suppress potential artifacts due to cable movements, since amplification first takes place at the electrode itself. Ground and reference electrodes were placed between Fz and FCz and between Cz and Fz, respectively. Participants were asked to relax face and jaw muscles and minimize eye blinks and swallowing during data recording to further limit EEG artifacts. Tri-axial accelerometers (Compumedics, Neuroscan, Singen, Germany) were placed over the L3 segment of the lumbar spine and on the medial side of both ankles to detect moment of heel strike and toe-off during the gait cycle and for gait analysis. EEG and accelerometer signals were recorded at a sampling rate of 512 Hz using a portable amplifier (Siesta, CompumedicsNeuroscan, Singen, Germany), synchronized with video recordings of all sessions and sent via WIFI to Profusion EEG-software (v. 5.0, Compumedics Neuroscan, Singen, Germany) on a laptop and stored for later analysis.

### Gait Analysis

Time-points of left and right heel strike and toe off were determined using the trunk accelerometer by an approach introduced by Sejdic et al. (2015) and are briefly summarized here. For the trunk accelerometer, the X, Y, and Z axes corresponded with medial/lateral, superior/inferior and anterior/posterior directions, respectively. In the first phase of the analysis, time points of maximum positive accelerometer amplitude in the Y direction were selected, which identified the different steps. The X direction was used to determine whether a step was made with the left or right leg. In the second phase, left and right toe off were identified, which were characterized by the first negative peak after the large positive peak in the Y direction that was selected in the first phase. Left and right heel strike were determined in the third phase, which were characterized by the first negative peak before the largest positive peak in the Z direction. These time points of left and right heel strike and toe off served as a marker for EEG analysis and were used to calculate gait characteristics for both conditions. Stride time was determined by calculating the time interval in seconds between two consecutive right heel strikes. Next, % double support phase and % swing phase were calculated by dividing the average time interval between consecutive heel strike and toe off and between toe off and heel strike, respectively, by the average stride time and multiplying this by 100. Stride time variability was determined by calculating stride time coefficient of variation, i.e., dividing stride standard deviation by the mean stride time. Swing time symmetry ratio was calculated as an index of gait symmetry. Here, the largest average swing time (either left or right) was divided by the smaller average swing time (either left or right) so that all individual values were > 1.0 where 1.0 denotes perfect symmetry. Kinovea video analysis software^1^ (version 0.8.15) was used to determine walking speed and step length. Walking speed was determined by dividing the length of the trajectory between two predetermined points (50.44 m) by the time it took the participants to complete this trajectory. Step length was calculated by dividing the length of this same trajectory by the number of steps needed to complete it. Both walking speed and step length were corrected for participant’s height.

### EEG Data Pre-processing and Analysis

MATLAB 2015a (The Mathworks, Inc., Natick, Massachusetts, United States) using EEGLAB 14_1_2b (sccn.ucsd.edu/eeglab) was used to perform pre-processing and analyses of the EEG data. EEG data were divided into the two different walking conditions and down sampled offline to 256 Hz to speed up computations. EEG recordings from these two walking conditions were truncated to straight line walking segments, i.e., starting, stopping and turning segments were removed. First, data was high pass filtered at 1 Hz using a finite impulse response filter with zero phase shift and line noise at 50 and 100 Hz was removed using the Cleanline technique (nitrc.org/projects/cleanline/). Next, channels that exhibited substantial artifacts were removed using the following criteria based on (Gwin et al., 2011): (1) channels with magnitude < 30 or > 10,000 μV; (2) channels with kurtosis > 5 standard deviations from the mean; (3) channels uncorrelated with the neighboring channels (*r* < 0.4) for more than 1% of the total time; (4) channels with standard deviation qualitatively higher than the other channels. Subsequently, EEG data were re-referenced to the average of the remaining channels, which was shown to minimize motion artifacts when performed as a post-processing step (Kline et al., 2015). Epochs were created from 1,000 ms before until 2,000 ms after time of right heel strike. Infomax independent component analysis was applied on the cleaned data set to temporally transform the EEG channel data into independent component signals. An equivalent current dipole model was computed using the DIPFIT function within EEGLAB, which best explained the scalp topography of each of these independent components. Next, independent components were excluded from the data set if the projection of the equivalent current dipole model to the scalp accounted for less than 80% of scalp map variance (Gwin et al., 2011) or when topography and time-course of the independent component was reflective of eye movement artifacts (Jung et al.,2000a,b). The remaining independent components were assessed and classified as electrocortical sources or muscle sources using their power spectra, ERSP and locations of their equivalent current dipoles. Independent components with spectral power peaks at stride frequency and broadband ERS and ERD were also removed, as these were thought to be primarily related to movement artifacts rather than electrocortical activity. Examples of such movement related or muscle related artifacts are shown in a previous paper by our group (Weersink et al., 2019). Finally, one last visual inspection was performed to confirm the quality of the cleaned data.

Afterward, the complete dataset was split into epochs from 1,000 ms before until 2,000 ms after right heel strike. ERSP was calculated for these epochs using the gain model, which is the default mode in EEGLAB (Delorme and Makeig, 2004). Event related spectral power changes were analyzed by the ERSP index:

$$E\,R\,S\,P\,\left(f,t\right)=\frac{1}{n}\,\sum_{k=1}^{n}{\left(F_{k}\,\left(f,t\right)\right)}^{2}$$

where for n trials (i.e., gait cycles), *F*_*k*_(*f*, *t*) is the spectral estimate of trial *k* at frequency *f* and time *t*. Individual ERSP results show group mean values for time-frequency points across the input epochs, where higher or lower spectral power differs from mean power during one gait cycle. Time points for gait events were aligned by time-warping single trial spectrograms of each subject and channel to the individual mean time interval between right heel strikes using a linear interpolation function available in the EEGLAB toolbox. Finally, condition group average ERSP plots for FC1, Fz, FC2, C3, Cz, C4, CP1, Pz, and CP2 were generated. To provide additional insight in the spatial distribution, 32 channel ERSP scalp distribution maps were made for both conditions for the 20–50 Hz frequency range during the four consecutive phases of the gait cycle. This frequency range was chosen because ERD-ERS alternations were most prominently observed for these frequencies and previous studies found that primarily these higher frequencies are involved in the higher order organization of gait (Wagner et al., 2012; Seeber et al., 2014; Weersink et al., 2019). In locomotor data, the periodicity of the gait cycle dominates the low-frequency spectral components (<8 Hz) of EEG data, and therefore these frequencies were disregarded.

### Statistical Analysis

Statistical analysis of subject and gait characteristics was performed in SPSS version 23 for Windows (IBM Japan Ltd., Tokyo, Japan). Histograms and Q-Q plots were examined to determine whether data distributions met the normality assumption. A paired *T*-test was used to determine significant differences between conditions for normally distributed data, stride time, double support and swing time, swing time symmetry ratio and stride time coefficient of variation. The sign test was used for non-normally distributed data to statistically compare the conditions, i.e., walking speed and step length. Afterward, *p*-values were corrected for multiple comparisons using the Bonferroni correction method. To visualize ERSP, significant differences from the baseline average gait cycle log spectrum were computed with a permutation method (Delorme and Makeig, 2004). Significant ERSP differences between conditions were identified using a non-parametric paired permutation method corrected for multiple comparisons using the False Discovery Rate (FDR) method available within EEGLAB 14_1_2b. Statistical comparisons between the two conditions was done by paired statistics, meaning that only the 19 participants walking in amble gait were statistically compared with themselves walking normally. For all statistical tests, an alpha level of 0.05 was assumed.

## Results

### Gait Characteristics

As is shown in Table 1, walking in amble gait resulted in a significantly increased step length (*p* = 0.002) and stride time (*p* = 0.001) and reduced walking speed (*p* = 0.007) compared to normal gait. Although the stride time coefficient of variation was increased with 58.2% in amble gait compared to normal gait, this did not reach significance after correcting for multiple comparisons (*p* = 0.224). There were no significant differences between normal and amble gait in the distribution of swing or double support phase or in swing symmetry.

**TABLE 1:** Spatiotemporal gait characteristics based on accelerometers and video recordings.

	Normal gait (*n* = 35)	Amble gait (*n* = 19)	*p*-value	*t*-value
Step length (m)	0.71 ± 0.07*	0.76 ± 0.08*	0.002*	
Walking speed (m/s)	1.31 ± 0.36*	1.21 ± 0.39*	0.007*	
Stride time (s)	1.13 ± 0.13	1.29 ± 0.15	0.001	−3.747
Double support phase (%)	12.69 ± 2.57	13.86 ± 4.43	1.000	−1.408
Swing phase (%)	36.82 ± 3.34	36.71 ± 4.30	1.000	−0.219
Swing symmetry	1.05 ± 0.06	1.04 ± 0.04	1.000	0.225
STCV (%)	4.12 ± 2.78	6.52 ± 3.45	0.224	−2.322

Walking speed and step length are depicted as median ± interquartile range, while other gait characteristics are depicted as mean ± standard deviation. To compare the conditions, paired statistics (*non-normally distributed: Sign test, normally distributed: T-test) were calculated for the 19 participants performing both normal and amble gait. P-values are adjusted after Bonferroni correction. STCV, stride time coefficient of variation.

### Event Related Spectral Perturbations

**FIGURE 1:**
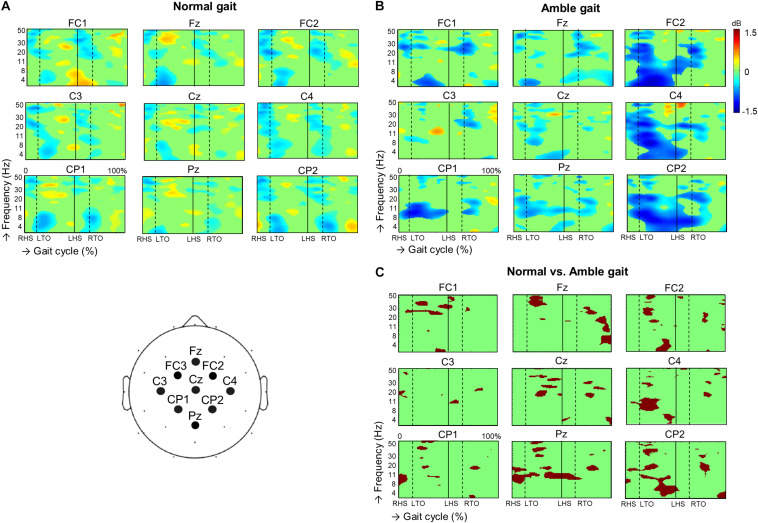
Dynamic changes across the EEG frequency spectrum from electrodes located over the sensorimotor region during the successive stages of the gait cycle during **(A)** normal gait (*n* = 35) and **(B)** amble gait (*n* = 19). For each condition, significant (*p* < 0.05) event related desynchronization is illustrated in blue and significant event related synchronization in red. Significant differences in ERSP between the two gait conditions were calculated for the 19 participants performing both normal and amble gait and is presented in **(C)**. Position of the EEG electrodes on the scalp is illustrated in the cartoon at the bottom left. ERSP, event related spectral perturbations; dB, decibel; RHS, right heel strike; LHS, left heel strike; RTO, right toe-off; LTO, left toe-off.

When visually comparing the ERSP plots of normal gait with those of amble gait (Figure 1), two differences stand out. Compared with normal gait, amble gait was associated with a less demarcated within-step alternating ERD/ERS pattern over all electrodes. Secondly, during amble gait a striking ERD was visible over the right hemisphere electrodes FC2, C4, and CP2 prior to and during the left swing phase.

Assessing these ERSP plots per electrode in more detail revealed that in the amble gait condition, the three frontal electrodes exhibited a significantly reduced high beta and low gamma ERS during especially the left swing phase (FC1 *p* = 0.0095, Fz *p* = 0.00049, FC2; *p* = 0.0045), which sometimes even turned into ERD. For example, particularly at Fz a strong ERS turned into a moderate ERD. It was further observed that ERD was significantly enhanced in the alpha and low beta frequencies at Fz (*p* = 0.0059) and FC2 (*p* = 0.0025) during the right and left swing phase, respectively.

Over the putative primary motor cortex, amble gait was also associated with significantly reduced beta/low gamma ERS during both swing phases, especially at the midline Cz electrode (*p* = 0.0015) and right C4 electrode (*p* = 0.0115), over the cortical representations of the legs and left arm area, respectively. When comparing amble with normal gait, the putative arm areas of the primary motor cortex, especially at the C4 electrode (i.e., the left arm representation in the right hemisphere) exhibited amble gait associated changes, including a significantly increased alpha/beta ERD during the left leg swing phase (*p* = 0.0085), which was accompanied by left forward arm swing in amble gait. The C3 electrode (i.e., the right arm representation in the left hemisphere) did not show such an obvious difference during the right leg swing phase, accompanied with right forward arm swing in amble gait. Only minor beta ERD reductions were seen during both double support phases (*p* = 0.0445). At all three parietal electrodes, ERD in the alpha/low beta range was significantly enhanced during both the double support and mid swing phases (CP1 *p* = 0.0205, Pz *p* = 0.0055, CP2 *p* = 0.0025). ERD enhancement in the high beta/low gamma frequency range was seen during the first half of the left swing phase over especially Pz (*p* = 0.0065) and CP2 (*p* = 0.0055).

**FIGURE 2:**
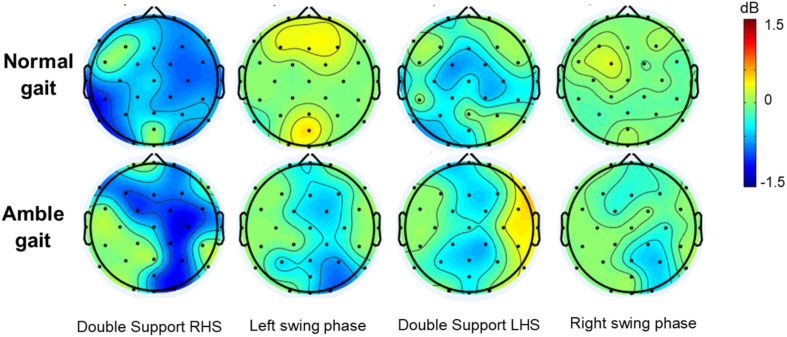
Group averaged topographic distribution of event related spectral perturbations (ERSP) over the entire scalp (32 electrodes) during normal (*n* = 35) and amble (*n* = 19) gait in healthy participants. Significant (*p* < 0.05) event related desynchronization is illustrated in blue and significant event related synchronization in red.

The spatial distribution of the characteristic high beta/low gamma alterations that occurred in the distinct double support and swing phases is further demonstrated by the ERSP scalp maps (Figure 2). These maps also illustrate that the alternating ERD-ERS pattern in this frequency range is diminished when participants walked in amble gait compared to normal walking, especially at electrodes located over the frontal areas. Additionally, the increased high beta/low gamma ERD in amble gait is indeed more lateralized in the right hemisphere, particularly the right frontal and parietal regions.

## Discussion

In the present study, we found characteristic differences in electrocortical activity between amble and normal gait. As both walking modes are characterized by antiphase movements of the arms as well as the legs, the re-ordering of this stereotypic movement pattern in amble gait provides insight in the dynamical qualities of cortical circuitry implicated in the control of antiphase movement patterns of opposite limbs in human gait. Cortical activity was recorded at the described electrodes. In the following discussion we refer to the putative cortical regions that generated this activity. We acknowledge that the recorded activity may result from a mixture of underlying sources, which implies that the observed effects cannot be unequivocally assigned to an exactly demarcated brain region.

When participants walked in a normal fashion, a regular within step ERD-ERS alternation was observed over the putative sensorimotor cortex and putative SMA, consistent with previous literature (Gwin et al., 2011; Wagner et al., 2012, 2014; Weersink et al., 2019). The more challenging amble gait condition resulted in a reduced demarcation of the within-step alternating ERD-ERS pattern over especially the right premotor area, right primary motor cortex, and (especially right) parietal areas. Here, a reduced ERS during the swing phases of the two legs was seen with an enhanced ERD prior to and during the left swing phase.

Over the putative SMA, such reduced ERD-ERS alternation was particularly due to the change of ERS into ERD in the first part of the left swing phase. One might infer that the switch from such prominent ERD-ERS alternation into gradual ERD fluctuation reflects a reduction of the optimal balance between movement initiation and inhibition across the two hemispheres, which is considered to be an important SMA contribution to regularly tuned antiphase movements of the opposite hands (Brinkman, 1981; Stephan et al., 1999; Potgieser et al., 2014). In a similar way, antiphase arm swing is thought to serve efficient gait control (Collins et al., 2009; Kuhtz-Buschbeck and Jing, 2012; De Graaf et al., 2019; Weersink et al., 2019, 2020). In the less efficient amble gait condition, antiphase movement of upper limbs is maintained, as in normal gait. However, different from the cyclic pattern of the opposite arms in overlearned normal gait, amble gait requires that the ipsilateral limbs remain in-phase. This walking mode may thus be seen as a more complex task, for which wider distributed circuitry is recruited, while involvement of the SMA is reduced.

Premotor and parietal regions, particularly in the right hemisphere, appeared to participate in such extended circuitry, given the strongly increased and prolonged ERD which was a prominent feature related to the reduced demarcation of the ERD-ERS pattern in these regions during amble gait. The contribution of these regions to increased movement complexity is consistent with previous reports of their involvement in organizing new or more complex patterns of coordination, including a less familiar phasing pattern similar to amble gait (Swinnen, 2002; Wenderoth et al., 2004). Increased activity of these regions has also been described in PD patients performing manual antiphase movements, which was accompanied by reduced SMA activity, when compared to healthy subjects (Wu et al., 2010). Such increase can be regarded to reflect recruitment of brain regions to compensate for dysfunction of the SMA and basal ganglia (Samuel et al., 1997; Wu et al., 2010, 2015). Increased interactions between the functionally impaired SMA in PD and posterior cortical regions that channel sensory information has also been argued to explain that these patients are more vulnerable to external stimuli in gait (van der Hoorn et al., 2014b). Similarity in the activation profiles of interconnected premotor and parietal regions fits their common contribution to neuronal networks serving the sensory guidance of movement (Wise et al., 1997; Rizzolatti et al., 1998), including enhanced attention to such sensorimotor transformations (Mengotti et al., 2020).

One might consider that in amble gait the conjoint antiphase movement of upper and lower limbs implies a strong shift of attention to the swing side of the body, which is not the case in normal gait. As such (covert) attention is equally focused on the alternating left and right swing side, one may infer that the observed right-hemisphere dominance of the associated ERSP alterations represents a distinct right-lateralized function implicated in these “spatial shifts of attention.” Such lateralization is consistent with right parietal-premotor dominance associated with spatial perceptual transformations in visuomotor control (Gitelman et al., 1996; Mattingley et al., 1998; De Jong et al., 1999; Mengotti et al., 2020). Right fronto-parietal brain activity related to the transitions from antiphase to in-phase patterns (Meyer-Lindenberg et al., 2002; Aramaki et al., 2006) further suggests a right hemisphere dominance in coordinating these phase patterns. The presence of such right-lateralized function is also supported by the increased involvement of specifically the right dorsal premotor cortex in antiphase compared to simple in-phase movement (Sadato et al., 1997; de Jong et al., 2002). As complex phase patterns require increased monitoring of afferent sensory information from the various limbs, it seems a logical consequence that enhanced ERD occurred in parietal regions during amble gait. Besides sensory feedback from the joints and muscles, processing of this movement also concerns feedforward processing concerning the sensory consequences of motor commands by mechanisms such as corollary discharge, in which the parietal and frontal areas are similarly involved (McCloskey, 1981; Sommer and Wurtz, 2008). In the context of covert attention, it is interesting to notice that within fronto-parietal networks, dorsal and ventral attention systems have been discerned (for review see Mengotti et al., 2020). The relation between amble gait and particularly a dorsally located system would be consistent with the contribution of such dorsal attention system to *monitoring* the ongoing spatial regularities of motor control, while a more ventrally located fronto-parietal network has been suggested to be more involved in processing (spatial) *expectations*, which is particularly relevant in circumstances of external changes (Mengotti et al., 2020).

In normal gait, the primary motor cortex directly drives muscle activity, which corresponds with the ERD prior to and during the actual movement (Petersen et al., 2012; Farrell et al., 2014; Artoni et al., 2017). We similarly found such ERD over the medial primary motor cortex, i.e., putative leg area, which was equally present during both double support phases, while the lateral primary motor cortex, i.e., putative arm area, exhibited ERD prior to both forward and backward swing phases. The alternating within-step ERD-ERS pattern over the putative primary motor cortex thus indicates that this normal gait pattern is driven per step. Particularly in the high beta and low gamma range, the putative SMA (at Fz) exhibited a similar pattern of alternation, consistent with its contribution to organizing step-related movement elements. The above discussed reduction of the alternating ERD-ERS pattern and prolonged strong ERD in both the right primary motor cortex and upstream areas of the right hemisphere, predominantly prior to and during the amble gait left leg swing phase is consistent with the notion that this more complex walking pattern is stride driven. As proposed above, a predominantly right-hemisphere contribution to higher-order motor control, at task level, may be regarded to represent such driving force that more generally overarches the entire gait cycle, beyond the fine-tuned step elements.

One may wonder whether such right-hemisphere function is also expressed in a behavioral dominance of left leg and left (forward) arm movement. We did not find such asymmetric swing phase of the legs in amble gait. However, the increased stride time variability, increased step length and reduced walking speed pointed at a generally reduced efficiency in amble gait, when compared to normal gait. In normal gait, studies on limb dominance remained contradictory and are difficult to extrapolate to amble gait (Sadeghi et al., 2000). The maintained symmetry of movement characteristics in amble gait adds an argument that the right-hemisphere ERD enhancement is not simply due to movement itself or limb dominance, but indeed represents a higher-order motor function serving coherent bilateral movement. Such function may possibly be associated with guiding perceptual information into the motor system of both hemispheres, in which the right dorsal premotor cortex has been proposed to play a specific role (van der Hoorn et al., 2014a).

Our study primarily focused on the cortical control of arm swing, with application of advanced EEG analysis. It is interesting, however, to mention a century-lasting debate to what extent gait-related arm swing is either actively controlled or simply a consequence of passive dynamics (for a review see Meyns et al., 2013). Some of the earliest studies on gait suggested that arm swing is purely passive, i.e., a consequence of thorax movements, gravity and inertia (Gerdy, 1829; Weber and Weber, 1836). More recently, a passive dynamics model with free-swinging arms has also been reported to possibly explain a major contribution of passive dynamics to both normal and amble gait with little effort gaining substantial energetic benefit (Collins et al., 2009), although amble gait induced a much greater reaction moment from the ground, requiring an active contribution of more active shoulder muscles in their *in vivo* study (Collins et al., 2009). Shoulder activity that drives arm swing has been widely recognized as a necessity for normal gait besides these passive components (Elftman, 1939; Fernandez Ballesteros et al., 1965; Hogue, 1969). Without this shoulder activity arm swing amplitude and relative phase would significantly decrease (Goudriaan et al., 2014), underscoring the importance of this active component for arm swing during continuous gait. Neuronal control of such shoulder muscle activity is largely organized by interconnected Central Pattern Generators (CPG) that play a pivotal role in generating this four limb locomotion pattern. However, these CPGs do not operate autonomously as higher order regulation of this interlimb coordination is concurrently achieved at brainstem and cortical level. Involvement of the motor cortex in this active control of gait related arm swing was previously confirmed using transcranial magnetic stimulation (Barthelemy and Nielsen, 2010). Another strong argument for such active cortical contribution to gait-related arm swing came from our recent study, demonstrating that upper and lower limb muscles receive input from a common cortical and subcortical driver during gait with shoulder muscle activity being able to drive and shape lower limb muscle activity (Weersink et al., 2021). In amble gait, EMG activity has previously been shown to be enhanced in flexor and extensor shoulder muscles, compared to such activity in normal gait (Kuhtz-Buschbeck and Jing, 2012). This might suggest suboptimal passive dynamics in amble gait, demanding more active components to be recruited from the wider distributed cortical circuitries, which is consistent with the EEG results of the present study. Overall, one may assume that both normal and amble gait patterns result from an interplay between both passive and active components, with the active component emerging from integrated cortical and subcortical pathways.

With regard to the experimental design, there were some limitations. The amble gait condition was performed at the participant’s preferred speed, which resulted in larger steps and a reduced walking speed. One might therefore question whether the observed EEG alterations in this condition are not simply related to these differences in lower limb gait characteristics. However, previous studies did examine the effect of different walking speed and step length on gait-related ERSPs (Wagner et al., 2016; Nordin et al., 2020). Although the experimental setting slightly differed between our study and the referred studies, their findings related to a reduced walking speed consisted of subtle changes over the entire cortex that remained step driven, whereas the changes related to our amble gait condition were primarily lateralized to the right hemisphere and became more stride driven. It is therefore unlikely that the observed effects are solely due to the altered lower limb characteristics. Besides, although participants practiced the amble gait condition before the recordings started, amble gait remained a novel task that requires the participant to pay more attention to their gait compared to normal gait, which could also have contributed to the observed ERSP findings. However, as our findings were also not comparable to previous ERSP observations during a novel gait task with normal anti-phase arm swing (walking synchronized with an auditory cue; Wagner et al., 2016), we believe that our findings are mainly related to the altered arm swing. Another limitation of the study is the limited spatial resolution of the EEG recordings. When interpreting condition-related differences in cortical activity recorded at the different EEG electrodes, it is important to keep in mind that, while located over a certain brain area, the recorded activity may result from a mixture of underlying sources. For this reason, the observed effects cannot unequivocally be assigned to a distinct single brain region. Future studies with more EEG channels, enabling higher spatial resolution are necessary to further identify contributions of specific brain areas to the observed effects.

## Conclusion

By employing the experimental condition of amble gait, we challenged cerebral circuitry underlying antiphase patterns of opposite limb movements in human walking. EEG and accelerometer data recorded during amble gait, compared to normal gait, demonstrated a shift from step- to more stride related power modulations in distinct EEG frequency bands, characterized by enhanced and prolonged ERD over especially the putative right premotor cortex, right primary motor cortex and (especially right) parietal areas. This indicated higher-order motor control implicated in this four-limb movement pattern, which was embedded in distributed networks upstream of the primary motor cortex and primarily lateralized in the right hemisphere. Given that the accompanying shift from prominent ERD-ERS alternation into gradual ERD fluctuation over the putative SMA reflected a reduced involvement of this medial frontal region in organizing step-related movement elements, one may consider the right hemisphere contribution complementary to that of the SMA in conditions of more complex antiphase movements. Such interaction between these medial and lateral cortical regions is consistent with the compensatory role of the latter in impaired SMA function seen in PD.

## Data Availability Statement

Since sharing data in an open-access repository was not included in our participant’s consent and therefore compromises our ethical standards, obtained data are only available on request from the corresponding author.

## Ethics Statement

The studies involving human participants were reviewed and approved by the Medisch Ethische Toetsingscommissie UMC Groningen. The patients/participants provided their written informed consent to participate in this study.

## Author Contributions

JW, BJ, and NM: conception, design, analysis, and interpretation. JW: data acquisition and first draft of the manuscript. BJ and NM: revising manuscript. All authors have read and approved the final version of this manuscript and agreed to be accountable for all aspects of the work in ensuring that questions related to the accuracy or integrity of any part of the work are appropriately investigated and resolved, designated as authors qualify for authorship, and all those who qualify for authorship are listed.

## Conflict of Interest

The authors declare that the research was conducted in the absence of any commercial or financial relationships that could be construed as a potential conflict of interest.

## Publisher’s Note

All claims expressed in this article are solely those of the authors and do not necessarily represent those of their affiliated organizations, or those of the publisher, the editors and the reviewers. Any product that may be evaluated in this article, or claim that may be made by its manufacturer, is not guaranteed or endorsed by the publisher.

## Footnotes

1
www.kinovea.org
